# Judging the possibility of the onset of diabetes mellitus type 2 from reported behavioral changes and from family history

**DOI:** 10.1186/s40842-022-00147-w

**Published:** 2023-01-11

**Authors:** María Teresa Muñoz Sastre, Paul Clay Sorum, Lonzozou Kpanake, Etienne Mullet

**Affiliations:** 1 University of Toulouse, CERPPS, Maison de La Recherche, 5 Allées Antonio Machado, 31058 Toulouse Cedex 9, France; 2 Albany Medical College, AMC Med-Ped, 1019 New Loudon Road, Cohoes, NY 12047 USA; 3 University of Québec - TELUQ, 5800 Rue Saint-Denis, Bureau 1105, Montréal, QC H2S 3L5 Canada; 4 Institute of Advanced Studies (EPHE), Paris, France; 5 Guadalajara, Spain

**Keywords:** Type 2 diabetes, Lay diagnosis, Judgment analysis

## Abstract

**Background:**

Undiagnosed type 2 diabetes is common and can lead to unrecognized health complications. Given that earlier detection can reduce the damage to vital organs, it is important for all persons to be able to make the connection between certain new manifestations in their bodies and the possibility of diabetes. This study examined the extent to which people use the behavioral changes they observe in others (or in themselves), as well as relevant family history, to judge the possibility of the onset of diabetes.

**Methods:**

One hundred and fifty-six adults living in France examined a set of realistic vignettes describing a person with (or without) signs suggestive of diabetes (e.g., increased thirst, family antecedents) and judged the possibility of the disease in each case.

**Results:**

Overall, 36% of participants focused on reported symptoms when judging the possibility of diabetes, 37% focused on family history, and 29% were not able to use the information or tended systematically to minimize the possibility of diabetes.

**Conclusions:**

People in France and probably around the world need a greater awareness not only of the factors putting them at risk of diabetes, but also of the specific signs and symptoms suggesting that they might be developing it.

## Background

Type 2 diabetes mellitus is a disease characterized by high levels of glucose in the blood due to a gradual malfunction in the physiological regulatory mechanisms. It is more common in the obese and the elderly and in some families. Perceived symptoms include a greater than usual thirst associated with frequent urination, weight loss despite persistent appetite, and transient vision problems. It tends to evolve, however, with no or minimal accompanying symptoms for several years [1]. Indeed, undiagnosed type 2 diabetes is common; it is estimated in 2017 to be 50% of those with diabetes globally, especially among disadvantaged groups and in lower income countries [2]. Since it can lead to unrecognized health complications, earlier detection can reduce the damage to vital organs [3]. It is, therefore, important for all persons to be attentive to its subtle manifestations, to report them accurately to their primary care physicians, and to be tested at the slightest doubt. This means that people need to be able to make the connection between certain new manifestations in their bodies and the possibility of diabetes.

Fukuoka et al. [4] examined lay knowledge of type 2 diabetes in a sample of healthy Californian adults. When asked to describe its signs and symptoms, only 45% were able to report at least one of them: increased thirst by 20%, increased urinary frequency by 15%, increased fatigue by 14%, weight change by 13%, dizziness by 5%, and vision problems by 4%. Kayyali and collaborators [5] conducted a similar study with adults living in London. While more than 65% of participants were able to identify increased thirst and urinary frequency as symptoms, a smaller proportion identified increased fatigue (58%), vision problems (49%), and weight change (46%) as symptoms. Related studies have also been conducted in developing countries [6–9].

The present study was conducted from a different, functional perspective. The question asked was not only whether participants recognize certain behavioral manifestations as symptoms of diabetes, but also what they do with this information when judging the possibility that a person is developing diabetes. In other words, to what extent can people use the behavioral changes reported by others (or observed in themselves), as well as relevant family history, to judge the possibility of the onset of diabetes?

A scenario technique was used. Various realistic situations describing a person with (or without) signs suggestive of diabetes–increased thirst, weight loss, vision disturbances, family antecedents–were created, and participants were asked to judge the possibility of the disease in each case. Given the diversity of responses observed in previous studies [4, 6], we expected that various subgroups of participants would be sensitive to different clues of the disease, i.e., that some participants would rely primarily on visible symptoms to judge and others would take more account of family history and age.

## Method

### Participants

Participants were 156 adults (53% women) aged 18 to 79 (*M* = 35.95, *SD* = 14.95). Their demographic characteristics are shown in Table 1. They were approached on the streets of several cities in the south of France by four research assistants specially trained for this type of survey. A total of 300 people were contacted: 52% agreed to participate.

**Table 1:** Demographic Characteristics of the Sample. Composition of the Clusters

	Cluster					
Variable	Never	Reported Changes	Family Antecedents	Vision Disturbances	Undetermined	Total
Gender						
Males	4 (6)	16(22)	25(34)	11(15)^a^	17(23)	73
Females	9(11)	26(31)	33(40)	4(5)^a^	11(13)	83
Age						
18–25 years	5(9)	14(24)	22(38)	7(12)	10(17)	58
26–40 Years	1(2)^a^	12(27)	18(40)	6(13)	8(18)	45
41 + Years	7(13)^a^	16(30)	18(34)	2(4)	10(19)	53
Education						
Secondary	8(14)^a^	12(21)	21(38)	5(9)	10(18)	56
College	2(3)^a^	22(33)	24(36)	5(7)	14(21)	67
University	3(9)	7(22)	13(41)	5(16)	4(12)	32
Know People with Diabetes						
No	4(6)	22(30)	25(34)	6(8)	16(22)	73
Yes	9(11)	20(24)	33(40)	9(11)	12(14)	83
Diabetic						
No	13(9)	42(27)	56(36)	15(10)	28(18)	154
Yes	0(0)	0(0)	2(100)	0(0)	0(0)	2
Total	13	42	58	15	28	156

Values in parentheses are percentages calculated across each row. Know Cases of Diabetes = Know people in the family or close friends who have developed diabetes. Values with the same superscript are statistically different, *p* < .05

### Material

The material consisted of 60 vignettes describing the situation of a woman who has various features suggestive of diabetes. A first set of 48 vignettes was obtained by orthogonally crossing the levels of five factors: (a) the person's age (about 23 or about 65 years old), (b) whether or not she has a more frequent need to drink (very often thirsty, more often thirsty than usual, no change), (c) whether or not she has lost weight (lost about four kilograms or no change in weight), (d) whether she has experienced any vision problems (frequent or no problems), and (e) a family history of diabetes known to her (several cases of diabetes in the family or no cases). A second set was obtained by replicating 12 vignettes from the main design and adding a Soreness factor (frequent joint soreness or not) that is not a symptom of early diabetes.

An example of the scenario is as follows: "For the past two months or so, Mrs. Paez, 64, has noticed that she is often thirsty, that she tends to drink a lot, and that the volume of her urine has, of course, increased. She has also noticed a significant weight loss (four kilos) even though she is not particularly dieting. In fact, she even seems to have a good appetite now. Finally, she has noticed that her vision is occasionally blurred. This lasts a while and then everything goes back to normal. She is well aware that there are several cases of diabetes in her family. Do you think Mrs. Paez could have diabetes?” The response scale was an 11-point scale with on the left "Definitely not" (0) and on the right "Definitely yes" (10).

### Procedure

Each volunteer was tested on an individual basis, in a quiet room, usually in the volunteer's home. Some participants were interviewed in an unused classroom at the university. The process followed Anderson's [10] guidelines for this type of study (see also Vera Cruz and collaborators [11]); i.e., participants were familiarized with the materials and how to use the response scale before examining the vignettes. The sequence of presentation of the vignettes differed between participants and was determined randomly. Participants took 25 to 45 min to complete the assessments. No one complained about the number of vignettes or their plausibility. The study was approved by the Ethics Committee of the University of Toulouse, France. Informed consent of all participants was secured orally, and full anonymity was ensured.

### Statistical analysis

A cluster analysis was performed on the raw data using the K-means method advocated by Hofmans and Mullet [12]. A five-cluster solution was retained because it produced the most interpretable findings. A first analysis of variance was performed on the initial set of data, using a Cluster x Age x Family History x Vision Problems x Weight Loss x Thirst, 5 × 2 × 2 × 2 × 2 × 3 design. A second analysis was performed on the complementary set of data, using a Cluster x Soreness x Vision Problems x Weight Loss x Thirst, 5 × 2 × 2 × 2 × 3 design. As, in both cases, the cluster effect and most of the interactions involving the cluster factor were significant at *p* < 0.001, subsequent analyses were performed at the cluster level. Complete results are available from the corresponding author.

## Results

The first cluster (*N* = 13, 8%, not shown) was called *Always Improbable* because the mean ratings observed were always close to the left end of the response scale (*M* = 2.33). Ratings were slightly higher when vision disturbances were present (*M* = 2.95) than when they were absent (*M* = 1.74), η^2^_p_ = 0.73. As can be observed in Table 1, the oldest participants (13%) and those with a college degree (14%) were more often members of this cluster than middle-aged participants (2%) or those with a university degree (3%).

**Fig. 1:**
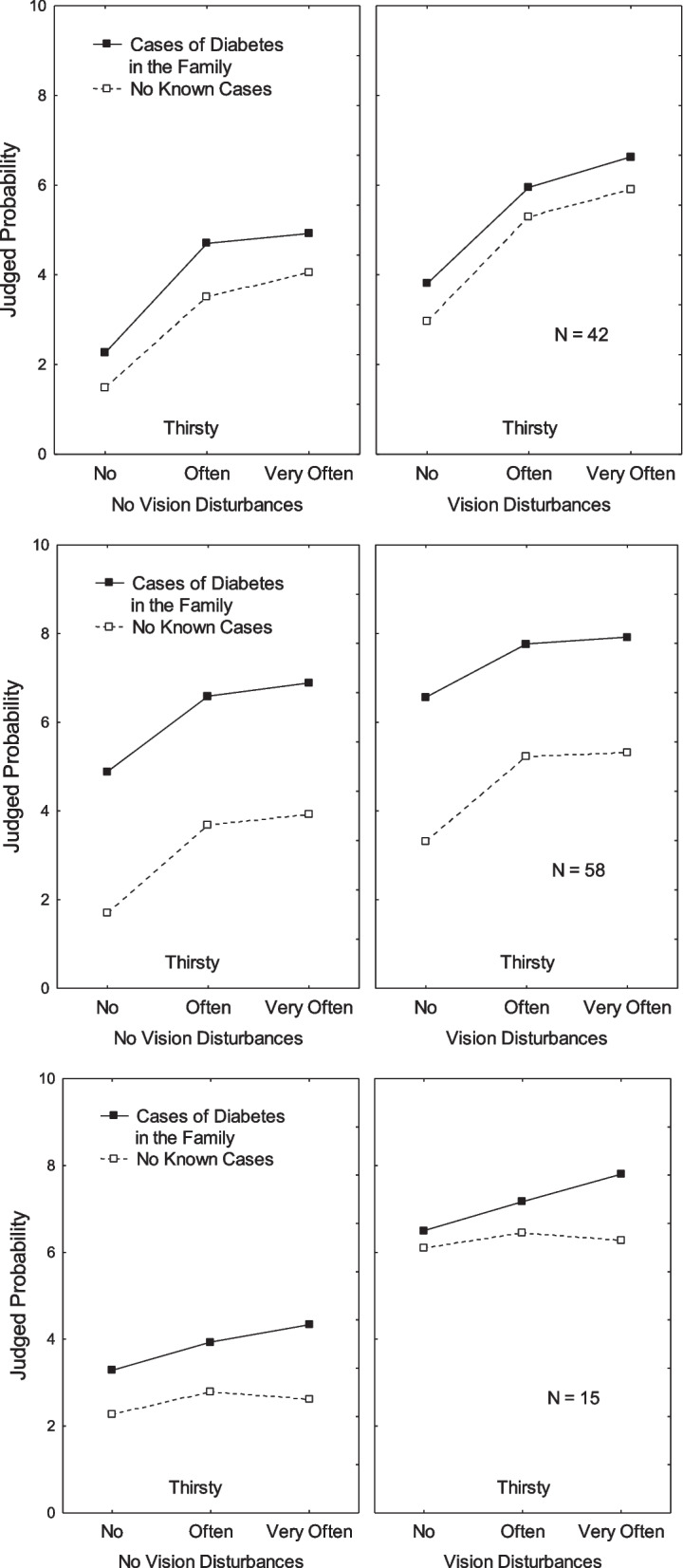
Judgements have been plotted along the vertical axis. The three levels of Thirst are on the horizontal axis. The two curves correspond to Family antecedents. Each panel correspond to one level of vision disturbance. Each row corresponds to a cluster: *Reported Symptoms* (top panels), *Family History* (center panels) and *Vision Disturbance* (bottom panels). Ratings have been pooled over the age and weight loss factors

The second cluster (*N* = 42, 26%) was called *Reported Symptoms* because vision disturbance, weight loss, and thirst had the strongest effects. As shown in Fig. 1 (top panels), ratings were higher when the person experienced vision disturbances (*M* = 5.09) than when she did not (*M* = 3.49), η^2^_p_ = 0.76, and when she experienced an increased need to drink (*M* = 5.38) than when she did not (*M* = 2.63), η^2^_p_ = 0.78. In addition, ratings were higher when she experienced weight loss (*M* = 5.17) than when she did not (*M* = 3.41), η^2^_p_ = 0.72.

The third cluster (*N* = 58, 37%) was called *Family History* because this factor had the strongest impact on ratings. As can be observed in Fig. 1 (center panels), ratings were considerably higher when known cases of diabetes existed in the family (*M* = 6.77) than when not (*M* = 3.86), η^2^_p_ = 0.84. In addition, ratings were, as in the second cluster, higher when the person experienced vision disturbances (*M* = 6.02) than when she did not (*M* = 4.61), η^2^_p_ = 0.66, when she experienced weight loss (*M* = 5.92) than when she did not (*M* = 4.72), η^2^_p_ = 0.58, and when she experienced an increased need to drink (*M* = 6.02) than when she did not (*M* = 4.12), η^2^_p_ = 0.68. As can be observed in Table 1, both participants with diabetes were members of this cluster.

The fourth cluster (*N* = 15, 10%) was called *Vision Disturbances* because this factor had the strongest impact on ratings. As can be observed in Fig. 1 (bottom panels), ratings were considerably higher when these disturbances were present (*M* = 6.72) than when they were not (*M* = 3.21), η^2^_p_ = 0.97. In addition, ratings were, as in the second cluster, higher when the person experienced weight loss (*M* = 5.58) than when she did not (*M* = 4.34), η^2^_p_ = 0.68, when she experienced joint soreness (*M* = 5.69) than when she did not (*M* = 4.59), η^2^_p_ = 0.38, and when there was a family history of diabetes (*M* = 5.50) than when not (*M* = 4.42), η^2^_p_ = 0.76. The impact of thirst was stronger when there was a family history of diabetes than when there was not, η^2^_p_ = 0.24. Male participants (15%) were more often members of this cluster than females (5%).

The fifth cluster (*N* = 28, 18%, not shown) was called *Undetermined* because the mean ratings observed were always close to the center of the response scale (*M* = 5.08). Ratings were slightly higher when the person was 65 years old (*M* = 5.69) than when she was younger (*M* = 4.47), η^2^_p_ = 0.31. No other factor had an impact on ratings.

## Discussion

This study of French adults’ judgments concerning the likelihood of diabetes had several findings. First, most participants used the pieces of information additively and correctly when making their judgments about the likelihood of diabetes, even if with a difference in emphasis. Some (36%) focused on reported symptoms. They did not ignore information about family history, but the importance attributed to it was secondary. Others (37%) focused on family history without, however, ignoring reported symptoms.

Second, only a minority of participants (18%) were not at all able to make use of the given information to infer a risk of diabetes. Another minority (8%) tended systematically to minimize the possibility of diabetes.

Third, in all cases, the person’s age (a relevant risk factor) and the presence of joint soreness (an irrelevant symptom) were hardly taken into account. People’s ability to distinguish between relevant and irrelevant factors needs further investigation.

Fourth, the participants’ cognitive performance was, overall, quite reassuring. The majority seem likely to be able, in the future, to recognize the key manifestations of type 2 diabetes and integrate them into a reasonable judgment of the likelihood of having it.

### Limitations

The first limitation of this study is that it is a convenience sample of lay people living in one region of France who were willing to complete a time-consuming judgment task. The second limitation is the relatively young age of most participants. Many of them were therefore unlikely to feel immediately at risk for type 2 diabetes, unlike their parents and grandparents. Future studies should further analyze the judgments of representative samples of older people, those most directly affected by the onset of this disease.

The third limitation is the small number of factors that could be studied. An orthogonal design requires a multiplicative increase in the number of scenarios as additional symptoms, such as the protagonist's current weight, are considered. Such an increase quickly becomes too burdensome for participants. Further studies could address a different set of symptoms, including the degree of obesity and actual level of exercise.

The fourth limitation is that, as in the other studies cited above, the participants had diabetes in mind when they evaluated persons’ symptoms. Future studies should examine what condition would spontaneously come to their minds when they are presented with these same scenarios without any prior hint about diabetes.

## Conclusions

Indeed, people around the world need a greater awareness not only of the factors putting them at risk of diabetes [13], but also of the specific signs and symptoms suggesting that they might be developing it. Accordingly, we urge the development and testing of educational programs, whether at the community or the primary care level, in which people are made aware not only of risk factors, as was accomplished in a public multimedia program in a region of China [14], but also of the signs and symptoms of type 2 diabetes. They must learn not just that diabetics exhibit these signs and symptoms but how to use this information to judge of the possibility, in the concrete circumstances of everyday life, that a person experiencing these signs and symptoms (or reporting them) may in fact be developing type 2 diabetes.

## Data Availability

All data collected is anonymous, is available and can be accessed by contacting the corresponding author.

## Abbreviations

M: Mean
N: Number
SD: Standard deviation
ANOVA: Analysis of variance
